# A protocol for a pooled analysis of cohort studies: The association between depression and anxiety in epileptic disorders

**DOI:** 10.1371/journal.pone.0295328

**Published:** 2023-12-07

**Authors:** Yan Wang, Changbo Shen, Junyan Zhang, Qingcheng Yang, Jianshe Li, Jun Tan, Hang Yu, Zubing Mei

**Affiliations:** 1 Department of Neurology, The Third Affiliated Hospital of Xinxiang Medical University, Xinxiang, China; 2 Department of Neurology, The People’s Hospital of Anyang City, Anyang, China; 3 Department of Neurology, Xinxiang Central Hospital, Henan Province, China; 4 Emergency Department, Changhai Hospital, Naval Military Medical University, Shanghai, China; 5 Department of Anorectal Surgery, Shuguang Hospital, Shanghai University of Traditional Chinese Medicine, Shanghai, China; 6 Anorectal Disease Institute of Shuguang Hospital, Shanghai, China; Xiamen University - Malaysia Campus: Xiamen University - Malaysia, MALAYSIA

## Abstract

**Background/Introduction:**

Depressive and anxiety disorders constitute major mental health challenges affecting adults of all ages globally. It has been reported that individuals with depressive or anxiety disorders face an elevated risk of developing neurological conditions, including seizures and epilepsy. Additionally, people with these disorders tend to exhibit distinct clinical outcomes compared to the general population. However, the associations between depressive or anxiety disorders and epilepsy remain contentious. Thus, this study aims to elucidate the associations between these neuropsychiatric disorders, including depressive and anxiety disorders, and epilepsy or seizures.

**Methods:**

We will systematically search three electronic databases—PubMed, EMBASE, and the Cochrane Library—from inception through March 2023 to identify relevant cohort studies investigating the associations between depressive or anxiety disorders and epilepsy or seizures. Two independent reviewers will extract data from eligible studies using pre-designed standardized data extraction forms, and cross-check results. A third author will resolve any discrepancies. Quality assessment will be performed using the Newcastle-Ottawa Quality Assessment Scale (NOS). Pooled risk estimates (Relative risks or hazard ratios with their 95% CI) will be calculated using the DerSimonian-Laird random-effects model. If between-study heterogeneity is identified, we will conduct subgroup analyses or meta-regressions to explore the possible sources of heterogeneity (participants, exposure, outcome, and study design) stratified by various study characteristics. Potential publication bias will be detected through the inspection of funnel plot asymmetry, complemented by the Egger linear regression approach (Egger’s test) and the Begg rank correlation test (Begg’s test).

**Discussion:**

This pooled analysis will evaluate the association between depressive or anxiety disorders and epilepsy or seizures, providing high-level evidence to inform early identification and prevention strategies for epilepsy or seizures.

**Ethics and dissemination:**

Given that the data utilized for analysis in this pooled analysis does not involve human subjects or medical records, no ethical approval is required for this study. We intend to present the results of this study at national or international conferences or submit the findings to a peer-reviewed journal.

**OSF registration number:**

DOI 10.17605/OSF.IO/WM2X8

## Introduction

Mental health disorders represent a significant cause of morbidity worldwide, with common conditions including depressive disorders, anxiety disorders (primarily generalized anxiety and social anxiety), and other stress or mental health-related disorders [1]. It is estimated that 10% of the global population currently lives with at least one mental disorder, and nearly half of the population may experience at least one mental disorder during their lifetime [2]. According to World Health Organization statistics, depressive disorder and anxiety disorder are among the most prevalent mental disorders globally, affecting approximately 5–6% of people worldwide and with lifetime prevalence rates reported to be as high as 30% respectively [3, 4].

Epilepsy, a prevalent neurological disease characterized by recurrent seizures, exhibits significant co-morbidity. Common co-existing conditions with epilepsy include psychiatric disorders, cognitive deficits, and migraines. The prevalence of these comorbidities often depends on factors such as age, seizure type, and epilepsy etiology [3]. Various pathophysiological processes, including inflammation, gliosis, ion channel dysfunction, and synaptic remodeling, have been implicated in the development of epilepsy[4]. Furthermore, it has been suggested that epilepsy and depressive disorders may share certain common pathophysiological mechanisms [5].

Previous research has suggested that individuals with depressive and anxiety disorders may face higher risks of developing neurological conditions, including seizures and epilepsy [6–8]. Additionally, this population may experience different clinical outcomes compared to control groups. For instance, Zachary et al. found that depressive disorders were strong risk factors for epilepsy in individuals without brain tumors [9]. Other studies have also reported an association between depressive disorders and an increased risk of developing epilepsy [10]. Furthermore, the severity of depressive disorders has been linked to the severity of epilepsy [10], and incident of epilepsy has been associated with elevated risk of developing depressive disorders [11]. The potential common pathophysiological mechanisms might provide insight into the heightened risk of developing epilepsy following incident of depressive disorders. Nevertheless, the relationships between depressive or anxiety disorders and epilepsy or seizures are under debate. As such, this study aims to establish the associations between neuropsychiatric disorders, including depressive and anxiety disorders, and epilepsy or seizures.

Given the increasing number of epidemiologic investigations exploring the associations between depressive or anxiety disorders and the risk of epilepsy or seizures, along with conflicting outcomes, a comprehensive systematic review of the available evidence is warranted for integration into clinical practice. Consequently, this review will address the following question: Do individuals with depressive or anxiety disorders have a higher risk of developing epilepsy or seizures compared to those without depressive or anxiety disorders?

## The review

### Standard protocol approvals, registrations, and patient consents

We will adhere to the Cochrane Handbook to conduct this systematic review, and the Preferred Reporting Items for Systematic Review and Meta-Analyses Protocol (PRISMA-P) (S1 Table) combined with the Meta-analysis of Observational Studies in Epidemiology (MOOSE) reporting guidelines to report this systematic review [12]. The protocol of this project has also been registered on the website of the open science framework (OSF) (https://osf.io/wm2x8/) under the registration number of DOI 10.17605/OSF.IO/WM2X8 [13].

### Search strategy and literature screening

We will perform a comprehensive literature search using medical subject headings (MeSH) or Entree terms as well as free-text keywords across three electronic databases (PubMed, EMBASE, and Cochrane Library) to identify cohort studies examining the associations between depressive or anxiety disorders and epilepsy or seizures. Articles published from the inception of each database until March 2023 will be considered. The detailed search strategies and specific search terms (Depressive Disorder or Depression or Antidepressive Agents or Anxiety or Anxiety disorders or generalized anxiety disorder or panic disorder or phobias or Anti-Anxiety Agents) and (Epilepsy or Seizures) and (cohort or longitudinal or follow-up or prospective or retrospective or database or population) will be employed. No language restrictions will be applied during the literature search. The detailed search strategies for PubMed, Embase, and Cochrane Library can be found in Tables 1–3. In addition to the electronic database search, we will manually examine the reference lists of retrieved citations to identify relevant publications, including systematic reviews, meta-analyses, and conference abstracts, in order to supplement our search results. When multiple publications involving the same study cohort are found, we will select the article with either the most recent or comprehensive data for extraction. A multi-step literature selection process will be employed, consisting of duplicate removal using EndNote software, an initial selection phase based on title and abstract review, and a subsequent full-text evaluation based on the established inclusion and exclusion criteria. Two independent investigators will carry out the screening process, with discrepancies resolved through discussion or consultation with a third investigator to reach consensus. Grey literature will not be included in the analysis due to the lack of peer review, which often poses a higher risk of bias. If necessary, we will contact co-authors or corresponding authors to obtain any missing or unreported data via email.

**Table 1:** Search strategy for Pubmed database.

***Antidepressant terms*:**
1 "Antidepressive Agents"[Mesh]
2 "Serotonin and Noradrenaline Reuptake Inhibitors"[Mesh]
3 "Serotonin Uptake Inhibitors"[Mesh]
4 “Neurotransmitter Uptake Inhibitors"[Mesh]
5 (antidepressant* or antidepressi* or serotonin uptake or serotonin reuptake or serotonin re-uptake or SSRI* or SNRI* or Agomelatin* or Alaprocat* or Bupropion or Citalopram or Desvenlafaxin* or DVS-233 or B2061014 or Duloxetin* or Escitalopram or Fluoxetin* or Fluvoxamin* or Milnacipran or Mirtazapin* or Paroxetin* or Reboxetin* or Sertralin* or Venlafaxin* or Vilazodon* or Levomilnacipran or Vortioxetin* or Lu AA21004 or Lu AA24530 or LY2216684 or Edivoxetin* or CX157 or Tyrima) [Title/Abstract]
6 or/1-5
***Depression terms*:**
7 "Depression"[Mesh]
8 "Depressive Disorder"[Mesh]
9 "Depressive Disorder, Major"[Mesh]
10. "Mood Disorders"[Mesh]
11. depress*[Title/Abstract]
12. or/7-11
***Anxiety terms*:**
13. "Anxiety"[Mesh]
14. "Anxiety Disorders"[Mesh]
15. "Agoraphobia"[Mesh]
16. "Panic Disorder"[Mesh]
17. (agoraphobi* or generalized or generalized or anxiety or separation anxiety or social anxi* or fear* or phobi* or mutism) [Title/Abstract]
18. 13-17/or
***Anti-Anxiety Agents terms***
19 "Anti-Anxiety Agents"[Mesh]
20. "Benzodiazepines"[Mesh]
21. "Butyrophenones"[Mesh]
22. "Phenothiazines"[Mesh]
23. "Monoamine Oxidase Inhibitors"[Mesh]
24. "Serotonin Agents"[Mesh]
25. "Histamine Agents"[Mesh]
26. "Barbiturates"[Mesh]
27. "Hypnotics and Sedatives"[Mesh]
28. "Adrenergic Agents"[Mesh]
29. (acebutolol or alimemazine or alprazolam or amitriptyline or atenolol or Lormetazepam or Loprazolam or Zopiclone or Chloral hydrate or Chlormethiazole) [Title/Abstract]
30. 19-29/or
31. 6 or 12 or 18 or 30
***Eplipsy or seizure terms*:**
32 "Epilepsy"[Mesh]
33 "Seizures"[Mesh]
34 (epilep* or seizure* or convuls*)[Title/Abstract]
35. or/20-22
***Study design terms*:**
36 "Retrospective Studies"[Mesh]
37 "Cohort Studies"[Mesh]
38"Longitudinal Studies"[Mesh]
39 "Follow-Up Studies"[Mesh]
40 "Prospective Studies"[Mesh]
41 (cohort or longitudinal or followup or prospective* or retrospective* or database* or population* or follow up)[Title/Abstract]
42 "Registries"[Mesh]
43 (registry or registries) [Title/Abstract]
44 or/17-24
***Final search results*: *Combining terms*:**
45 31 and 35 and 44

**Table 2:** Search strategy for Embase database.

***Antidepressant terms*:**
1 ’antidepressant agent’/exp
2 ’serotonin noradrenalin reuptake inhibitor’/exp
3 ’serotonin uptake inhibitor’/exp
4 ’neurotransmitter uptake inhibitor’/exp
5 (antidepressant* or antidepressi* or ‘serotonin uptake’ or ‘serotonin reuptake’ or ‘serotonin re-uptake’ or SSRI* or SNRI* or Agomelatin* or Alaprocat* or Bupropion or Citalopram or Desvenlafaxin* or DVS-233 or B2061014 or Duloxetin* or Escitalopram or Fluoxetin* or Fluvoxamin* or Milnacipran or Mirtazapin* or Paroxetin* or Reboxetin* or Sertralin* or Venlafaxin* or Vilazodon* or Levomilnacipran or Vortioxetin* or LY2216684 or Edivoxetin* or CX157 or Tyrima):ab.ti.
6 or/1-5
***Depression terms*:**
7 ’depression’/exp
8 ’major depression’/exp
9 ’mood disorder’/exp
10. 7-9/or
11. depress*:ab,ti
12. 10 or 11
***Anxiety terms*:**
13. ’anxiety’/exp
14. ’anxiety disorder’/exp
15. ’agoraphobia’/exp
16. ’panic’/exp
17. (agoraphobi* or generalized or generalized or anxiety or ‘separation anxiety’ or ‘social anxi*’ or fear* or phobi* or mutism):ab,ti
18. 13-17/or
***Anti-Anxiety Agents terms***
19 ’anxiolytic agent’/exp
20. ’benzodiazepine derivative’/exp
21. ’butyrophenone derivative’/exp
22. ’phenothiazine derivative’/exp
23. ’monoamine oxidase inhibitor’/exp
24. ’serotonin receptor affecting agent’/exp
25. ’histaminergic receptor affecting agent’/exp
26. ’barbituric acid derivative’/exp
27. ’hypnotic sedative agent’/exp
28. ’adrenergic receptor stimulating agent’/exp
29. (acebutolol or alimemazine or alprazolam or amitriptyline or atenolol or Lormetazepam or Loprazolam or Zopiclone or ‘Chloral hydrate’ or Chlormethiazole): ab,ti
30. 19-29/or
31. 6 or 12 or 18 or 30
***Eplipsy or seizure terms*:**
32 ’epilepsy’/exp
33 ’seizure’/exp
34 (epilep* or seizure* or convuls*):ab,ti
35. or/20-22
***Study design terms*:**
36 ’retrospective study’/exp
37 ’cohort analysis’/exp
38 ’longitudinal study’/exp
39 ’follow up’/exp
40 ’prospective study’/exp
41 (cohort or longitudinal or followup or prospective* or retrospective* or database* or population* or ‘follow up’): ab,ti
42 ’register’/exp
43 (registry or registries): ab,ti
44 or/17-24
***Final search results*: *Combining terms*:**
45 31 and 35 and 44

**Table 3:** Search strategy for Cochrane Library.

***Antidepressant terms*:**
#1 MeSH descriptor: [Antidepressive Agents] explode all trees
#2 MeSH descriptor: [Serotonin and Noradrenaline Reuptake Inhibitors] explode all trees
#3 MeSH descriptor: [Serotonin Uptake Inhibitors] explode all trees
#4 MeSH descriptor: [Serotonin Uptake Inhibitors] explode all trees
#5 ((antidepressant* or antidepressi* or serotonin uptake or serotonin reuptake or serotonin re-uptake or SSRI* or SNRI* or Agomelatin* or Alaprocat* or Bupropion or Citalopram or Desvenlafaxin* or DVS-233 or B2061014 or Duloxetin* or Escitalopram or Fluoxetin* or Fluvoxamin* or Milnacipran or Mirtazapin* or Paroxetin* or Reboxetin* or Sertralin* or Venlafaxin* or Vilazodon* or Levomilnacipran or Vortioxetin* or Lu AA21004 or Lu AA24530 or LY2216684 or Edivoxetin* or CX157 or Tyrima)):ti,ab,kw (Word variations have been searched)
#6 #1 or #2 or #3 or #4 or #5
***Depression terms*:**
#7 MeSH descriptor: [Depression] explode all trees
#8 MeSH descriptor: [Depressive Disorder] explode all trees
#9 MeSH descriptor: [Depressive Disorder, Major] explode all trees
#10 MeSH descriptor: [Mood Disorders] explode all trees
#11 (depress*):ti,ab,kw (Word variations have been searched)
#12 #7 or #8 or #9 or #10 or #11
***Anxiety terms*:**
#13 MeSH descriptor: [Anxiety] explode all trees
#14 MeSH descriptor: [Anxiety Disorders] explode all trees
#15 MeSH descriptor: [Agoraphobia] explode all trees
#16 MeSH descriptor: [Panic Disorder] explode all trees
#17 ((agoraphobi* or generalized or generalized or anxiety or separation anxiety or social anxi* or fear* or phobi* or mutism)):ti,ab,kw (Word variations have been searched)
#18 #13 or #14 or #15 or #16 or #17
***Anti-Anxiety Agents terms***
#19 MeSH descriptor: [Anti-Anxiety Agents] explode all trees
#20 MeSH descriptor: [Benzodiazepines] explode all trees
#21 MeSH descriptor: [Butyrophenones] explode all trees
#22 MeSH descriptor: [Phenothiazines] explode all trees
#23 MeSH descriptor: [Monoamine Oxidase Inhibitors] explode all trees
#24 MeSH descriptor: [Serotonin Agents] explode all trees
#25 MeSH descriptor: [Histamine Agents] explode all trees
#26 MeSH descriptor: [Barbiturates] explode all trees
#27 MeSH descriptor: [Hypnotics and Sedatives] explode all trees
#28 MeSH descriptor: [Adrenergic Agents] explode all trees
#29 ((acebutolol or alimemazine or alprazolam or amitriptyline or atenolol or Lormetazepam or Loprazolam or Zopiclone or Chloral hydrate or Chlormethiazole)):ti,ab,kw (Word variations have been searched) 8385
#30 #19 or #20 or #21 or #22 or #23 or #24 or #25 or #26 or #27 or #28 or #29
***Eplipsy or seizure terms*:**
#31 MeSH descriptor: [Epilepsy] explode all trees
#32 MeSH descriptor: [Seizures] explode all trees
#33 ((epilep* or seizure* or convuls*)):ti,ab,kw (Word variations have been searched)
#34 #31 or #32 or #33
***Study design terms*:**
#35 MeSH descriptor: [Retrospective Studies] explode all trees
#36 MeSH descriptor: [Cohort Studies] explode all trees
#37 MeSH descriptor: [Longitudinal Studies] explode all trees
#38 MeSH descriptor: [Follow-Up Studies] explode all trees
#39 MeSH descriptor: [Prospective Studies] explode all trees
#40 ((cohort or longitudinal or followup or prospective* or retrospective* or database* or population* or follow up)):ti,ab,kw (Word variations have been searched)
#41 MeSH descriptor: [Registries] explode all trees
#42 ((registry or registries)):ti,ab,kw (Word variations have been searched)
#43 #35 or #36 or #37 or #38 or #39 or #40 or #41 or #42
***Final search results*: *Combining terms*:**
#44 #6 or #12 or #18 or #30
#45 #34 and #43 and #44

### Eligibility criteria

Inclusion criteria will be established using the PECOS (population, exposure, comparison, outcome, study design) framework based on the criteria of the Cochrane Handbook for Systematic Reviews as summarized below [14].

### Population

This pooled analysis will include studies involving adult participants (aged ≥18 years) who have been previously diagnosed with depressive or anxiety disorders. Diagnosis of these disorders will be established instruments such as the using the 14-item Hospital Anxiety and Depression Scale (HADS), or criteria from the International Classification of Diseases (ICD-8, ICD-9, or ICD-10), or the Diagnostic and Statistical Manual of Mental Disorders (DSM), which includes generalized anxiety disorder, panic disorder, and phobias. Eligible participants should have no history of epilepsy or seizures or be diagnosed with epilepsy and seizures at the time of study enrollment in accordance with the ICD-9 or ICD-10 criteria for the study cohort [15–17].

### Exposure

The primary exposure of interest is defined as depressive or anxiety disorders, assessed using the 14-item HADS, ICD-8, ICD-9 or ICD-10 criteria, or DSM criteria.

### Comparisons

Included studies will compare the outcomes of interest between the exposed group (those with depressive or anxiety disorders) and an unexposed population (individuals with no history of depressive or anxiety disorders).

### Outcome

Eligible studies must report the outcome and provide accompanying risk estimate. The primary outcome under investigation is the risk of developing epilepsy or seizures in individuals with depressive or anxiety disorders, compared to those without such disorders.

### Study design

Prospective or retrospective population-based, community-based, or hospital-based cohort studies will be considered eligible, regardless of the language of publication.

### Exclusion criteria

1) Non-cohort study designs: Case-control studies, cross-sectional studies, case reports, or case series.

2) Insufficient or unclear data on exposure (depression and/or anxiety) and/or outcomes (seizure or epilepsy-related outcomes).

3) Studies with a primary focus on other psychiatric disorders or overlapping symptoms without clear distinction or stratification of depression and anxiety-related outcomes.

4) Animal studies or in vitro studies.

5) Review articles, editorials, commentaries, or meta-analysis.

6) Studies with duplicate or overlapping data.

### Identification of studies

#### Study selection

Two authors will independently conduct the study selection following a systematic process. The first step involves removing duplicate records, after which both investigators will review all titles and abstracts. Citations that do not meet the inclusion criteria will be subsequently excluded. The full texts of potentially relevant studies will then be assessed for eligibility based on the predefined inclusion criteria.

To address discrepancies and reach a consensus on all inclusion decisions, team meetings will be convened for discussion. If necessary, a senior co-investigator will be consulted to help resolve any disagreements. Additionally, we will perform a kappa agreement test to assess the concordance of literature selection results among the different investigators [18].

### Data extraction

We will create a standardized, piloted data extraction form to collect pertinent information from the included studies on study design, population, exposure, comparisons, and outcomes. Any disagreements between the review investigators will be resolved through discussion. The following variables will be extracted from each study: the first author, publication year, study design, study period and region, observation period, age at depressive or anxiety disorder diagnosis, control population, method of diagnosis for depressive or anxiety disorders, primary findings, and risk estimates for the association between epilepsy and seizures with depressive and anxiety disorders (Table 4).

**Table 4:** Variables included in the data abstract form.

Variables
• Study author
• Publication year
• Study design
• Study setting
• Study period
• Observation period
• Study region or country.
• Mean or median age of the study population
• Sex ratio of the study population
• Comorbid conditions of the study population
• Method or criteria of diagnosis for depressive or anxiety disorders
• Comparisons
• Cohort sample size
• Number of outcome events
• Follow-up period
• Primary outcome
• Estimates of the association for the study outcome

### Risk of bias

The risk of bias will be independently evaluated by two investigators using the Newcastle-Ottawa Scale (NOS) tool [19]. Two investigators will independently evaluate the methodological quality and risk of bias for each eligible cohort study based on 9 domains. A score of 7 or more on the NOS tool will be considered as low risk of bias [20, 21]. Discrepancies will be resolved through discussion between the investigators.

### Data synthesis

All statistical analyses in this meta-analysis will be performed using Stata Software Version 12.0 (Stata Corporation, College Station, TX, USA) and R (R version 3.6.1, R Foundation for Statistical Computing, Vienna, Austria) employing the "meta" package for meta-analysis. Either directly reported or calculated hazard ratios (HR) or relative risks (RR) along with their respective 95% confidence intervals (95% CI) will serve as risk estimates for dichotomous variables. In instances where studies do not report the RR or HR of epilepsy or seizures, alternative risk measures such as the incidence rate ratio (IRR) or standardized incidence ratio (SIR) will be selected to approximate the RR in comparisons between individuals with depressive and anxiety disorders and those who are disease-free or from the general population. Given the low prevalence of epilepsy and seizures in the general population, it is considered acceptable to pool relative risk, hazard ratio, IRR, and SIR together using meta-analysis methods [21]. Generally, fully adjusted relative risks will be chosen to summarize risk estimates. If the included studies do not provide relative risks or hazard ratios directly for associations between depressive and anxiety disorders and the risk of epilepsy or seizure outcomes, they will be calculated indirectly using the information in the original reports and previously described methods [22].

For continuous outcome data [such as depression severity measured on the Hamilton Depression Rating Scale (HDRS) or anxiety levels measured on the Hamilton Anxiety Rating Scale (HARS)], the weighted mean difference (WMD) will be utilized [23] when all studies share the same measurement tool and unit; otherwise, the standardized mean difference (SMD) will be calculated along with its corresponding 95% CIs for data synthesis. The Mantel-Haenszel method and inverse variance method [26] will be employed to pool dichotomous outcome data and continuous outcome data, respectively [24]. When there is evidence of between-study heterogeneity, the random-effects model using the DerSimonian and Laird method will be applied; in the absence of such heterogeneity, the fixed-effect model implementing the Mantel-Haenszel method will be used [25]. The I^2^ statistic (with an I^2^ value exceeding 50% denoting substantial heterogeneity) and the P value for heterogeneity (Cochrane Q test, where P < 0.10 indicates substantial heterogeneity) will be utilized to assess between-study heterogeneity, which represents the percentage of variation across studies attributable to between-study heterogeneity rather than chance [26, 27].

We will conduct subgroup analyses to investigate the sources of heterogeneity by categorizing studies based on study design (retrospective or prospective cohort study), study setting (population-based, community, or hospital-based), study center (single or multiple centers), geographic region (by continent), study period (before or after 2010), diagnostic methods for depressive or anxiety disorders (ICD criteria or others), sample size (≥ mean/median or < mean/median), mean age, control group (general population or with a history of past mental illness diagnoses), whether matched for crucial variables such as age and gender (yes or no), and risk of bias (high NOS quality or low NOS quality). Furthermore, univariate meta-regression analyses will be utilized to examine potential subgroup effects. We will perform a leave-one-out sensitivity analysis by sequentially excluding each study from the meta-analysis, recalculating the risk estimates for the remaining studies to assess the robustness of the summary estimates.

Publication bias will be evaluated by visually inspecting the symmetry of a funnel plot, where an asymmetric plot may indicate potential publication bias. We will further assess publication bias using Begg’s rank correlation test [28] and Egger’s weighted linear regression test [29] if there are at least 10 studies included. A P value below 0.05 for either Begg’s or Egger’s test indicates significant publication bias. In cases where missing studies are identified or publication bias is detected, we will employ Duval’s trim-and-fill method to adjust for publication bias by including the apparently missing studies [30]. All superiority statistical tests are two-sided and P values less than 0.05 will be considered statistically significant.

### Patient and public involvement

We will not involve patients or public in this study because it is a systematic review and meta-analysis using previously published data from related studies. The results of the present study will be disseminated through institutional or individual websites, peer-reviewed journals, international scientific meetings, networks, and social media.

### Ethical considerations

Since all the data we will use for analysis in this systematic review and meta-analysis does not involve human subjects or medical records, this study does not require ethical approval. We will determine the associations between the psychological disorders (including depression and anxiety disorders) and epilepsy or seizure outcomes. We will prepare a manuscript regarding the results of this systematic review and will present it at a national or international conference or submit it in a peer-reviewed journal.

### Amendments

This protocol for this systematic review and meta-analysis will also be updated and amended when necessary during the review process.

## Discussion

### Expected principal findings

The relationships between neuropsychiatric disorders, including depressive and anxiety disorders, with epilepsy remain a subject of debate. Through the integration of contemporary evidence from published observational cohort studies, our meta-analysis aims to offer consolidated risk estimates of the impact of depressive and anxiety disorders on the incidence of epilepsy or seizures. Over the past two decades, there has been a notable increase in the prevalence of neurological disorders, such as epilepsy and seizures, among individuals experiencing depressive and anxiety disorders [31–33]. Consequently, the urgency for early interventions to minimize the occurrence of these interrelated neurological disorders has grown. By elucidating the links between these conditions, our study may provide high-quality evidence for the relationship between these diseases.

### Potential study implications

This study addresses a critical question warranting investigation: Do individuals with depressive or anxiety disorders have an elevated risk of epilepsy or seizures compared to those without these disorders? Although numerous findings have emerged in recent years, the current state of knowledge in this area lacks decisive and persuasive evidence [34–36]. By conducting a comprehensive meta-analysis of cohort studies, we strive to shed light on the potential associations between these mental conditions and the risk of epilepsy or seizures, ultimately contributing to a better understanding of the interconnections between these disorders.

### Study strengths

The current study boasts numerous methodological strengths. First and foremost, our study aims to present the most contemporary and comprehensive analysis of the association between depressive or anxiety disorders and epilepsy or seizures published to date. Second, we will rigorously follow the Cochrane Handbook as well as PRISMA or MOOSE guidelines for conducting and reporting this pooled analysis [14]. In addition, the quality of each selected study will be assessed using the NOS quality criteria, allowing for an objective evaluation of the pooled evidence across domains [19]. Third, we will conduct several subgroup analyses, stratified by study baseline characteristics and coupled with meta-regression, to thoroughly investigate sources of between-study heterogeneity and establish the robustness of pooled results for each outcome. Moreover, a sensitivity analysis will be performed to verify the robustness and reliability of our findings. We will also examine publication bias and implement the trim-and-fill procedure to determine if missing studies could potentially influence pooled analysis results [37]. Lastly, we have designed and included detailed search strategies for each major online database (Tables 1–3) without imposing date or language restrictions. This approach aims to maximize the number of relevant articles obtained from a global context, mitigate potential publication bias on pooled results, and enhance the transparency and replicability of our findings [38].

### Study limitations

Our present study will inevitably have some potential limitations, which we believe should be acknowledged in future research. First, significant between-study heterogeneity is anticipated, especially with regards to epilepsy or seizures. This may be partly attributable to differences in baseline characteristics of the included studies, including study design and setting, demographic characteristics (age and sex) of participants, diagnostic criteria for depressive or anxiety disorders, outcome ascertainment for epilepsy or seizures, and adjusted confounders. Although we plan to conduct multiple analyses to adjust the results, additional inherent confounding factors may remain uncontrolled. Therefore, we urge that our results are interpreted with caution. Second, as our analysis will primarily be based on observational cohort studies, it would be inappropriate to infer a direct causal relationship between depressive or anxiety disorders and epilepsy or seizures [39]. The nature of the observational study design itself prohibits such extrapolation. To clarify potential direct links between these two conditions, further large-scale cohort or controlled clinical studies must be conducted. Third, our meta-analysis will rely on study-level data, as individual patient-level data will not be available for more detailed analyses. Lastly, we acknowledge that the exclusion of grey literature or unpublished data may introduce publication bias into our study. However, we will perform thorough searches in major databases, such as PubMed, Embase, and Cochrane Library, which collectively encompass most of the relevant journals concerning the subject worldwide [40]. Despite these limitations, our study-level pooled analysis aims to offer a comprehensive quantitative examination of the relationships between depressive or anxiety disorders and epilepsy or seizures.

### Future directions

Our study has several implications for future research. Firstly, the findings of our pooled analysis may prompt further investigation into the underlying mechanisms that link depressive and anxiety disorders with epilepsy and seizures. Understanding these mechanisms could lead to the development of targeted interventions and treatments. Secondly, future studies could explore the impact of different subtypes of depressive and anxiety disorders on the risk of epilepsy or seizures, as well as the potential role of comorbidities and confounding factors. Additionally, longitudinal studies could provide valuable insights into the temporal relationship between these conditions and help identify potential causal pathways. Finally, the inclusion of diverse populations and the consideration of potential cultural and socio-economic factors could enhance the generalizability of the findings and provide a more comprehensive understanding of the associations between these disorders.

### Clinical implications and practical value of the study

The synthesis of quantitative evidence from this study will provide updated and comprehensive insights into whether depressive or anxiety disorders are potential risk factors for the subsequent development of epilepsy or seizures. These findings may alert neurologists to the importance of early intervention in cases of depressive or anxiety disorders in order to prevent adverse events, such as the emergence of epilepsy or seizures. Furthermore, the identification of individuals at higher risk for epilepsy or seizures due to depressive or anxiety disorders could inform personalized treatment strategies and improve patient outcomes.

## Conclusion

In conclusion, our study contributes to the existing literature by providing a comprehensive analysis of the associations between depressive and anxiety disorders with epilepsy and seizures. The findings of this pooled analysis have the potential to impact clinical practice by raising awareness of the interconnections between these conditions and emphasizing the importance of early intervention and targeted management strategies. Further research is warranted to elucidate the underlying mechanisms and explore additional factors that may influence these associations.

## Supporting information

S1 Table PRISMA-P checklist. (DOCX)

## Abbreviations

CI: confidence interval
HADS: Hospital Anxiety and Depression Scale
ICD: International Classification of Diseases-8
IRR: incidence rate ratio
MDD: Major Depressive Disorder
Mesh: medical subject headings
MOOSE: the Meta-analysis of Observational Studies in Epidemiology
NOS: Newcastle-Ottawa Scale
PRISMA-P: the Preferred Reporting Items for Systematic Review and Meta-Analyses Protocol
RR: relative risk
SIR: standardized incidence ratio
SMD: standardized mean difference
WMD: weighted mean difference
